# Hydrochemistry of sediment pore water in the Bratsk reservoir (Baikal region, Russia)

**DOI:** 10.1038/s41598-021-90603-x

**Published:** 2021-05-27

**Authors:** V. I. Poletaeva, E. N. Tirskikh, M. V. Pastukhov

**Affiliations:** Vinogradov Institute of Geochemistry SB RAS, 1A Favorsky str., Irkutsk, 664033 Russia

**Keywords:** Environmental chemistry, Environmental sciences

## Abstract

This study aimed to identify the factors responsible for the major ion composition of pore water from the bottom sediments of the Bratsk water reservoir, which is part of the largest freshwater Baikal-Angara water system. In the Bratsk reservoir, the overlying water was characterized as HCO_3_–Ca–Mg type with the mineralization ranging between 101.2 and 127.7 mg L^−1^ and pore water was characterized as HCO_3_–SO_4_–Ca, SO_4_–Cl–Ca–Mg and mixed water types, which had mineralization varying from 165.9 to 4608.1 mg L^−1^. The ionic composition of pore waters varied both along the sediment depth profile and across the water area. In pore water, the difference between the highest and lowest values was remarkably large: 5.1 times for K^+^, 13 times for Mg^2+^, 16 times for HCO_3_^−^, 20 times for Ca^2+^, 23 times for Na^+^, 80 times for SO_4_^2−^, 105 times for Cl^−^. Such variability at different sites of the reservoir was due to the interrelation between major ion concentrations in the pore water and environmental parameters. The major factor responsible for pore water chemistry was the dissolution of sediment-forming material coming from various geochemical provinces. In the south part of the reservoir, Cl^−^, Na^+^ and SO_4_^2−^ concentrations may significantly increase in pore water due to the effect of subaqueous flow of highly mineralized groundwater.

## Introduction

In contrast to natural water, reservoir basins are specific water bodies formed under the influence of both natural and technogenic factors, which lead to irreversible changes in their hydrological, hydrochemical, and hydrobiological regimes^1^. The addition of technogenic organic and inorganic matter, which enters the reservoir with wastewater, surface runoff, and atmospheric deposition from contaminated areas, could bring about even more undesirable negative changes^2,3^. Similar to a natural pool, geochemical cycles in the reservoir are defined by a constant interaction between the abiotic and biotic components of the biosphere. However, water flow slows down and changes in sedimentation conditions after river flow regulation^4^ form a new ecosystem, in which the migration behaviour of elements will significantly change. In aquatic bodies, element geochemical cycles can be better understood through studies of the chemical composition of pore water that fills spaces between solid particles of bottom sediments^5,6^. Elemental composition of the pore water trapped in sediments can be used to study the conditions of matter circulation and its exchange between the bottom sediments and overlying water^7,8^. Pore water chemistry has proved to be a sensitive indicator of diagenetic transformations of bottom sediments, even when changes in their chemical characteristics may not be noticeable^9^. As was shown earlier, the initial composition of the pore water trapped in sediments is affected by some factors, such as cation exchange reactions between the pore water and bottom sediments, pH, and redox potential values, and the role of pore water in the formation of minerals^10^.

The Baikal-Angara water system includes Lake Baikal, which contains 20% of the world’s fresh surface water^11^, and the Angara River, the only channel of the lake’s surface runoff. Its resources are widely used for different purposes, such as drinking, household, and industrial water supplies in large cities and small settlements located on its shores. The creation and functioning of the cascade of Angara River reservoirs (Irkutsk, Bratsk, Ust-Ilimsk, Boguchan) led to radical changes in the hydrological, hydrochemical, and hydrogeochemical regimes of the Angara River^12,13^. The technogenic substances entering the Angara River and its water reservoirs have been found to affect the chemical composition of abiotic and biotic components^14,15^. Amongst the cascade reservoirs, the Bratsk, the second of the cascade, whose filling was completed in 1967, is considered the most technogenically stressed. Despite the great amount of information concerning the elemental composition of water^16,17^, bottom sediments^18,19^, and biota^20^ of the Bratsk reservoir, pore water chemistry remains an insufficiently studied geochemical sector. The pore water studies performed in the 1980s during the initial period of reservoir operation^21^ included only the determination of main cations (Na^+^, K^+^, Ca^2+^, Mg^2+^) and biogenic components (NO_3_^–^, SiO_2_^–^, PO_4_^3–^) in the pore solution. Since data on the chemical composition of pore water from sediments accumulated in the Bratsk reservoir over a 50-year operation period is very scarce, it is difficult to describe geochemical cycling in its ecosystem. Therefore, the main aims of the present study were (1) to determine concentrations of major ions in the sediment pore water and (2) to study the factors affecting pore water chemistry. As sedimentation conditions and factors influencing the geochemical environment in artificial reservoirs are different from those of natural basins, the study of sediment pore water in the Bratsk Reservoir is of particular relevance given the possibility of applying the obtained results and conclusions to other large reservoirs.

## Research area

**Figure 1:**
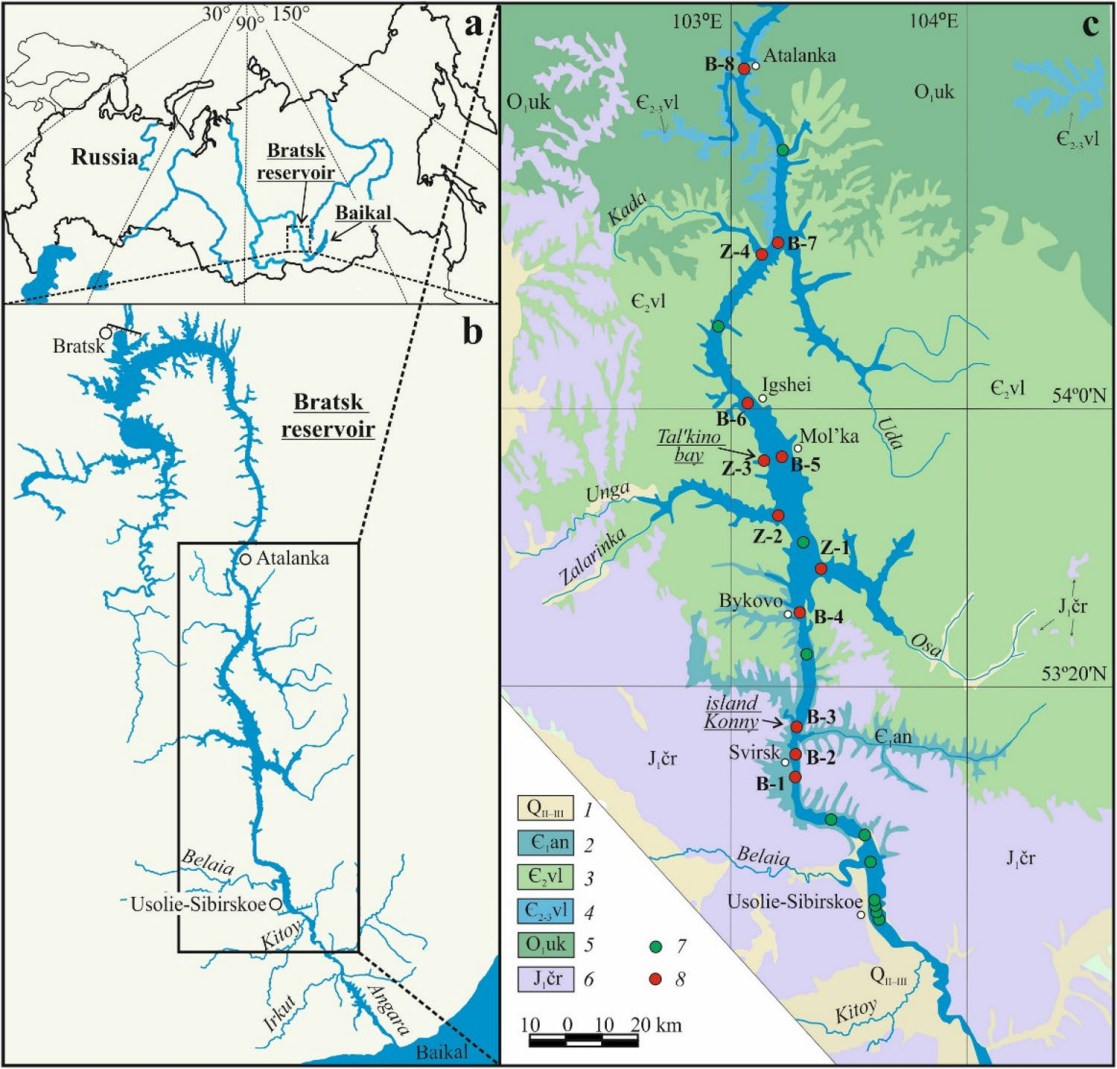
(**a**) Location of the Bratsk reservoir. (**b**) View of the wider study area. (**c**) Schematic geologic map with indicated sampling locations. Schematic maps, showing the locations within the studied area and geology structure of the area under study are taken from a digital catalog and are freely accessible (http://geo.mfvsegei.ru). Figure was created using CorelDRAW (21.3.0.755, www.corel.com). (**c**) 1—alluvial deposits (sand, pebbles, sandy loam); 2—lower Cambrian Angara formation (dolomites, anhydrite-dolomites, gypsum, rock salt); 3—Middle Cambrian Upper Lena formation (sandstones, siltstones, marls, mudstones, gypsum); 4—Middle-Upper Cambrian Vilyu formation (sandstones, siltstones, marls); 5—Lower Ordovician Ust-Kut formation (siltstones, mudstones, sandstones); 6—Jurassic sediments, Cheremkhovo formation (conglomerates, sandstones, siltstones); 7—sampling locations of overlying water; 8—sampling locations of overlying and pore water.

The Bratsk reservoir, a result of damming the Angara River due to the construction of the Bratsk Hydroelectric Plant, is located in the Baikal region of Russia (Fig. 1). It is one of the largest reservoirs in the world: its full storage capacity approximates 170 km^3^, the water surface area is equal to 5470 km^2^, the length is over 500 km, the maximum depth exceeds 150 m, and the maximum width is 25 km. The intra-annual water level fluctuations over the period of many years do not exceed 2–3 m. However, in prolonged low-water periods, the amplitude of water level fluctuations can be as high as 10 m^22^.

The water inflow into the Bratsk reservoir is dominated by surface inflow, which is generated from water input passing through the Irkutsk hydroelectric plant, located upstream, and a lateral inflow. At the gate of the Bratsk Hydroelectric Power Plant, the share of Baikal runoff is 62%^22^. When rivers and streams flow into the reservoir, they form numerous bays, creating a highly indented coastline. According to hydrological conditions in the reservoir, variable (from Usolie-Sibirskoe town to Svirsk town) and permanent (downstream Svirsk town) backwater effect zones can be recognized.

The change in the hydrological regime of the Angara River due to the creation of the Bratsk reservoir led to radical changes in its sedimentation characteristics. In the riverbed part of the reservoir, sedimentation is irregular, which can be explained by changes in water flow velocity, bottom relief, and the presence of eroded coastal ledges^12^. The highest sedimentation rates were found at the site where river conditions transformed into the reservoir ones. Here, more suspended material and more sediments accumulate due to a drop in the transport capacity of the water flow^23^. The thickness of the sediments decreases further downstream. The principal sediment-forming source in the Bratsk reservoir is the product of shore abrasion. The petrographic and lithological-geochemical composition of rocks composing the drainage area resulted in a vertical belt zoning of sedimentation conditions^24^. Annually, about 220 million tons of sedimentary material are brought into the reservoir due to shore abrasion, which accounts for about 97% of the total input^24^. In the upper part of the reservoir, the main sedimentary sources also include solid river runoff.

The reservoir lies along the southern part of the middle Siberian plateau in a meridional direction from south to north. The area under study is mainly composed of Lower Cambrian gypsum-carbonate-salt-bearing, Lower Paleozoic red-colored terrigenous-carbonate, and Upper Paleozoic–Mesozoic coal-bearing terrigenous formations, which constitute the sedimentary cover of the southern Siberian platform (Fig. 1c). The thickness of the Quaternary deposits is insignificant. They overlap the between-river massifs and fill up river valleys, shores, and floor of folds. The most common soils here are podzolic, sod-podzolic, gray forest, and chernozems^21^.

In terms of hydrogeology, the area under study is a part of the Angara-Lena artesian basin. In the area, there are several aquifers: groundwater in Quaternary sediments, fissure-stratal groundwater associated with the Jurassic, Silurian, Ordovician, and Cambrian rocks, and karst-fissure water in the Cambrian gypsum and carbonate rocks^12^. Feeding sources for groundwater are atmospheric precipitation and water inflow from faults and cracks.

## Methods

Taking into account different sedimentation conditions, the chemical composition of the pore water was studied in sediment retrieved from 12 sampling locations throughout the Bratsk reservoir (from Svirsk town to the Atalanka settlement) (Fig. 1, Table 1). Sampling sites B-1, B-2, and B-3 were located in the reservoir’s part, with the highest sedimentation rates typical of the riverbed section. Sampling sites B-4, B-5, B-6, B-7, and B-8 were situated in the area, marked by low sedimentation rates. Z-1, Z-2, Z-3 and Z-4 sampling locations were localized in the mouth of the reservoir’s entry to bays. The overlying water (a total of 50 samples) was collected from the board of a research vessel using an Ocean Test 110A water sampler from two horizons: surface (from a depth of 0.6 m) and bottom (in a metre layer from the bottom). The overlying water samples were taken from the stations lying from Usolie-Sibirskoe town to the Atalanka settlement. Sediment cores were sampled using a gravity sampler (GOIN, Russia) with plastic tubes of 1 m length and a return upper valve. The sediment cores for Eh and pH measurements in the sediment pore water were collected with a plastic tube with side holes for inserting electrodes. In all collected cores, the uppermost sediments (0.5–1 cm thick layer) were brown-orange. The underlying sediments included gray and dark gray silts. At seven locations, the obtained sediment cores contained flooded soil.

**Table 1:** Characterization of sediments and pore water sampling sites.

Sampling site	Location	Reservoir depth, m	Length of sediments (of flooded soil), cm	Sampling site	Location	Reservoir depth, m	Length of sediments (of flooded soil), cm
B-1	3 km upstream Svirsk town	11	30	B-7	Between Kada and Uda bays	33	7 (9)
B-2	1 km downstream Svirsk town	11	41	B-8	Atalanka settlement	45	14 (18)
B-3	Konny Island	12	86	Z-1	Entry to Osa Bay	18	26 (20)
B-4	Bykovo settlement	13	20	Z-2	Entry to Unga Bay	16	32 (10)
B-5	Mol’ka settlement	22	17 (17)	Z-3	Tal’kino Bay	12	20 (50)
B-6	Igzhei settlement	25	17	Z-4	Kada Bay	18	16 (8)

Retrieved sediment cores were placed within a nitrogen box. The sediment cores were opened and cut into segments (from 6 to 25 cm, depending on the core length) using plastic disposable utensils. The collected samples were placed into special plastic containers with tight-fitting lids for centrifuging. Pore waters were extracted in the field laboratory station by centrifuging the sample (Beckman centrifuge) for 30 min at 3000 rpm. Upon centrifuging, the centrifuge containers were opened in a nitrogen atmosphere. The pore water samples were filtered with plastic disposable syringes through a 0.45 μm membrane (acetate) filter and placed into preliminarily prepared tubes. The lids were additionally insulated with paraffin tape. The pore water samples were placed in a refrigerator and delivered at a temperature of + 4 °C for chemical analysis.

The pH and redox potential (Eh) of overlying water were measured in situ using an Eh/pH meter (HANNA HI98121), which features 0.01 pH and 1 mv Eh resolution. The pH of the sediment pore water was defined using Expert-001, Russia pH/Eh meter having pH-electrode ESK-10616 with 0.01 pH resolution. The Eh was measured in moist dark-gray sediments using an Expert-001 pH/Eh meter with Eh-electrode EPR-105, 1 mV Eh resolution.

The major ions in the water samples were determined at the Center for Collective Use "Isotope-Geochemical Research" of the IGC SB RAS (Irkutsk, Russia). The analysis was performed in accordance with a standard certified analytical quality control procedure. Reagent blanks and certified reference materials were used to control the analytical accuracy. In the overlying and pore water samples, flame emission spectrophotometry was used in the analysis of Na^+^ and K^+^ (PND F 14.1:2:4.138-98), atomic absorption spectrometry was applied to analyze Ca^2+^ and Mg^2+^ (PND F 14.1: 2:4.137-98), turbidimetric method was used to measure SO_4_^2−^ (PND F 14.1:2.159-2000), and titrimetric method was employed in the analysis of HCO_3_^−^ (GOST 31957-2012 (A.2)). Mercurimetric method was applied to study Cl^−^ in pore water samples (PND F 14.1:2.111-97) and overlying water (RD 52.24.402-2011).

The granulometric analysis was carried out by the pipette method using a semi-dispersed sample preparation process (boiling the ground sample in the presence of ammonia) (GOST 12536-79).

## Results

### Hydrochemical characteristic of overlying water

The overlying water was low-mineralized (TDS from 101.2 to 127.7 mg L^−1^); pH referred to water being either neutral or slightly alkaline (pH from 7.41 to 8.49). Concentrations of major ions in the water of the Bratsk reservoir channel part are presented in Figure S1 (Supporting information) and Table 2. The concentrations of the major ions in the overlying water taken from the Bratsk reservoir were found to be similar to those from Lake Baikal (Table 2). However, the reservoir samples had increased Cl^−^ and SO_4_^2−^ concentrations (Fig. S1). Notably, higher Cl^−^, SO_4_^2−^ and Na^+^ levels were found in the Angara River before its damming^29^. Higher concentrations of those ions can be explained by the distribution of the Cambrian deposits occurring downstream of Usolie-Sibirskoe town (Fig. 1c).

**Table 2:** Comparison of major ion concentrations (mg L^−1^) in overlying and pore waters of the Bratsk reservoir and other reservoirs.

	HCO_3_^−^	Cl^−^	SO_4_^2−^	Ca^2+^	Mg^2+^	Na^+^	K^+^	References
**Bratsk reservoir**								
Overlying water	66.4–83.0 ^a^ 71.9	< 1.0–4.5 3.0	< 10.0–13.7 11.3	17.6–22.8 19.8	3.3–4.5 3.9	2.4–5.1 4.0	1.0–1.1 1.0	This study
Sediment pore water	31.7–497.7	2.9–303.9	12.7–1022.0	26.0–522.0	5.1–68.3	7.7–183.0	2.1–11.1	
SD^b^	113.3	80.0	248.8	99.1	17.6	50.4	1.9	
Flooded soil pore water	38.1–370.9	4.1–285.6	54.0–2871.0	35.4–708.1	7.5–286.2	10.1–465.0	1.6–14.0	
SD	109.3	100.4	1158.0	289.7	105.5	179.7	4.1	
**Ivan’kovskoe reservoir, Russia**								
Overlying water	165	7	13	52.4	12.4	10.6		^25^
Pore water	140–512	14–28	6–57	55–131	11–22	5–21	3–12	^26^
**Lake Baikal**								
Overlying water	65.1	0.5	5.3	16.0	3.0	3.4	1.0	^27^
Pore water	< 150	< 2	< 9	< 28	< 4	< 5	< 1	^28^

^a^Minimum–maximum value in the numerator, mean value in the denominator; ^b^standard deviation.

Major ion concentrations were increased in the vicinity of Usolie-Sibirskoe town (Fig. S1). The concentrations of anions and cations were even much higher on the left bank of the reservoir close to Usolie-Sibirskoe town due to the supply of wastewater enriched in Cl^−^, SO_4_^2−^, Na^+^, Ca^2+^ and Mg^2+^ (see Supporting information, Table S1).

**Figure 2:**
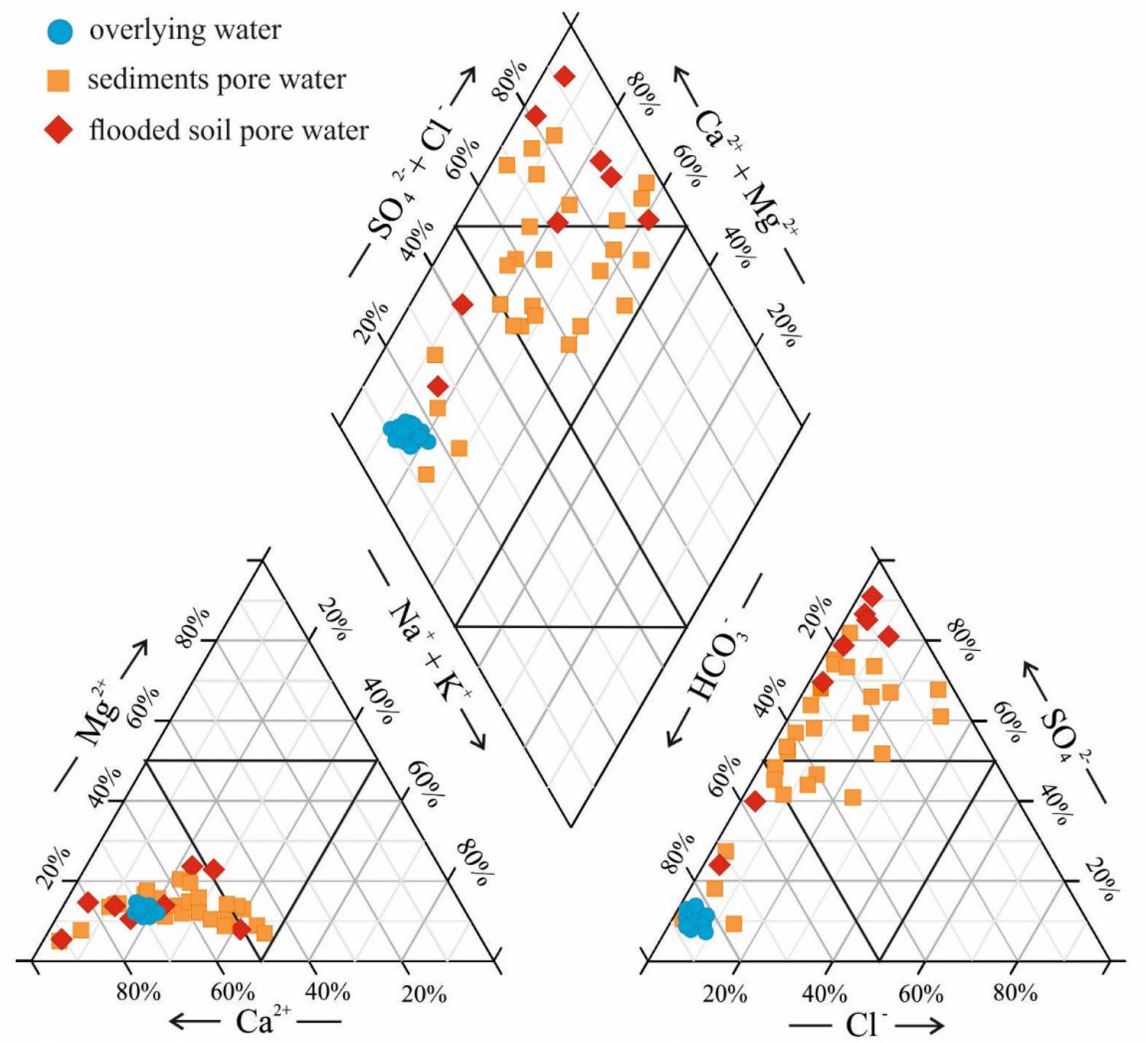
Piper diagram of reservoir pore water and overlying water. Figure was created using Grapher (17.2.435, www.goldensoftware.com) and CorelDRAW (21.3.0.755, www.corel.com).

Piper diagram is widely used for water type classification and characterization^30,31^; it enables identifying the hydrochemical facies of natural water. As shown by the Piper diagram (Fig. 2), in all overlying water samples taken from the Bratsk reservoir, the anions were dominated by HCO_3_^−^ and the cations were dominated by Ca^2+^. The diagram shows that the water type was primarily HCO_3_–Ca–Mg.

### Hydrochemical characterization of pore water in sediment

Significant spatial variations were observed with respect to mineralization: the pore water mineralization varied from 165.9 to 2073.0 mg L^−1^ in bottom sediments, and from 196.3 to 4608.1 mg L^−1^ in flooded soils. The pH value varied from slightly acidic (6.33) to slightly alkaline (7.88). In bottom sediments, Eh values ranged from − 73 to − 346 mV, decreasing at most sampling sites with depth. In flooded soils, the Eh values varied from − 145 to − 289 mV.

**Figure 3:**
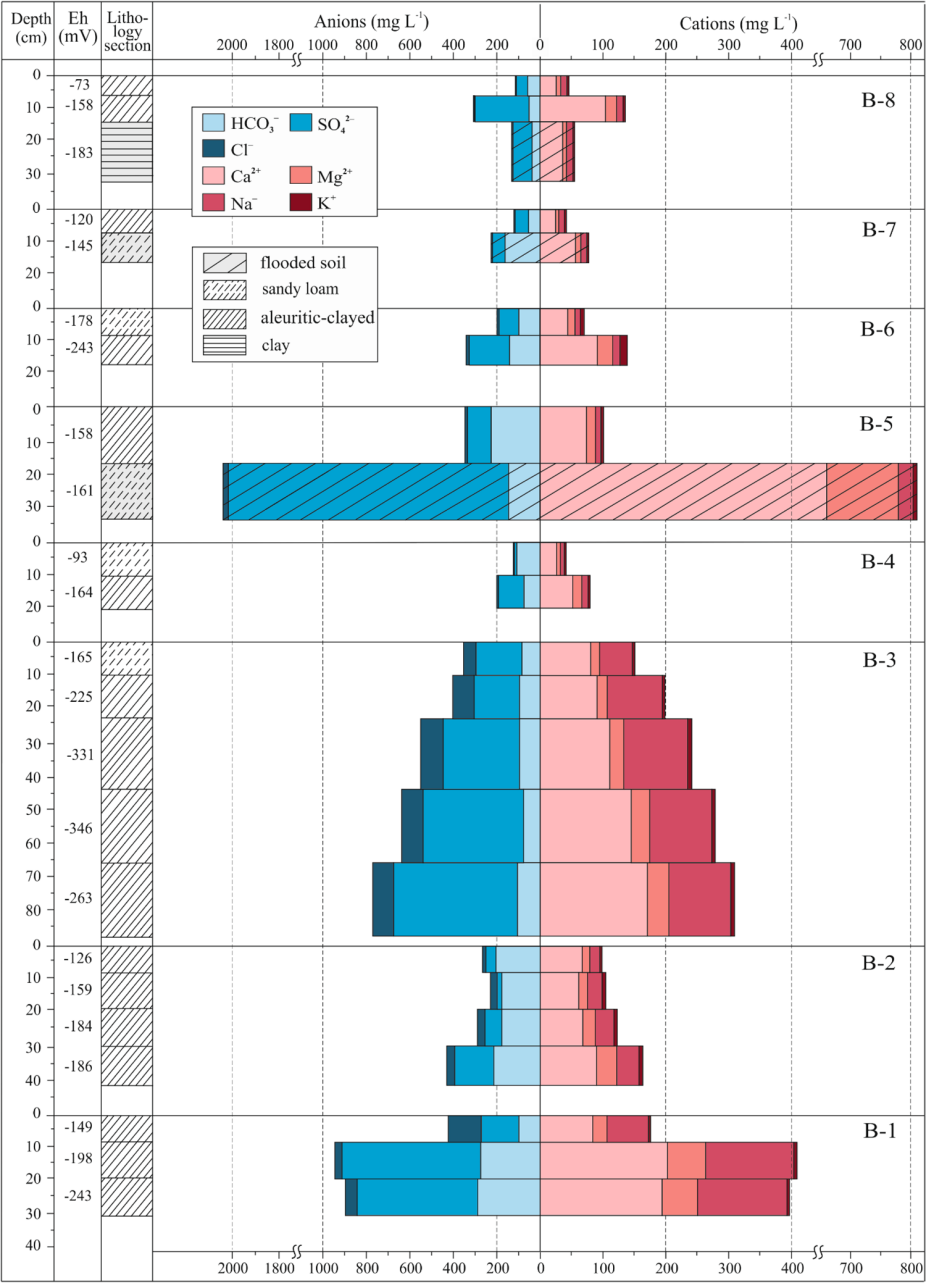
Major ion distributions in pore water from the channel part, Bratsk reservoir. Figure was created using CorelDRAW (21.3.0.755, www.corel.com).

**Figure 4:**
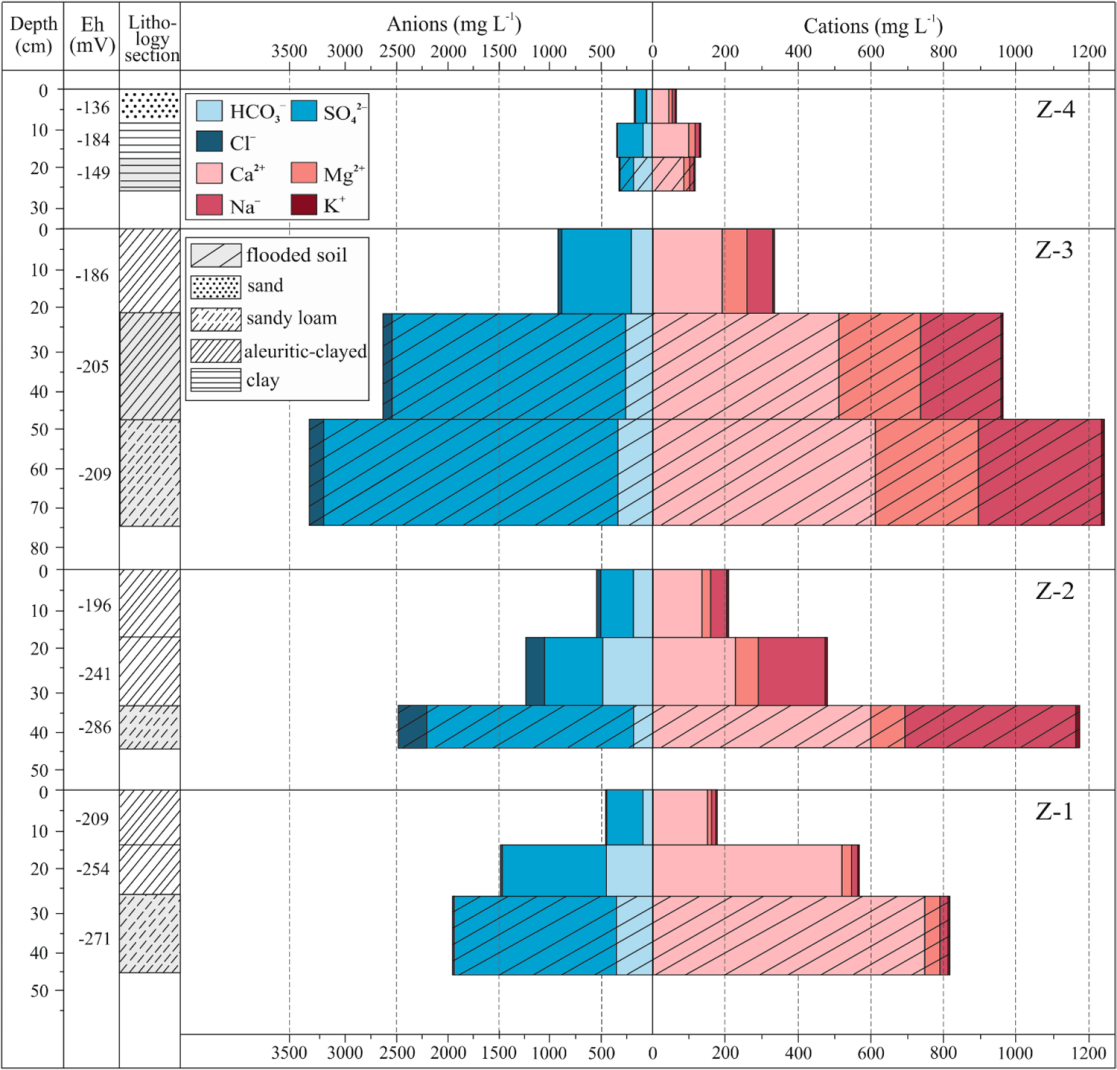
Major ion distributions in pore waters from entry to bays, Bratsk reservoir. Figure was created using CorelDRAW (21.3.0.755, www.corel.com).

Table 2 illustrates significant variability in concentrations of Cl^−^, SO_4_^2−^, Ca^2+^, Mg^2+^, Na^+^ ions in the pore water samples, which is indicated by high standard deviation. Note that the variations in the ionic composition were observed both across the water area and along the sediment and flooded soil depth profile (Figs. 3, 4). In relation to SO_4_^2−^ and Cl^−^ contents, HCO_3_^−^ concentrations were higher in the pore water samples throughout the B-2 (134.2–228.9 mg L^−1^) and B-5 (246.4 mg L^−1^) cores as well as in the samples collected from the first sediment layers of the B-4 (106.9 mg L^−1^) and B-6 (102.5 mg L^−1^) cores. Furthermore, relative to Cl^−^ levels (2.9–9.7 mg L^−1^), SO_4_^2−^ contents were higher throughout the B-5 core (95.5 mg L^−1^) and in samples taken from the first layers of the B-4 (12.7 mg L^−1^) and B-6 (92.4 mg L^−1^) cores. The samples collected from the first, third, and fourth layers of the B-2 core had higher SO_4_^2−^ (from 51.1 to 192.0 mg L^−1^) concentrations as compared with Cl^−^ levels (15.3–38.8 mg L^−1^). In the pore water samples taken from the second layer of the B-2 core, Cl^−^ contents (34.2 mg L^−1^) were higher in relation to SO_4_^2−^ levels (22.3 mg L^−1^). Looking at the distribution of major ions along the sediment depth profiles, it can be concluded that in most pore water samples, the levels of SO_4_^2−^ were higher than those of HCO_3_^−^ and Cl^−^. They included the samples taken throughout B-1 (180.8–651.2 mg L^−1^), B-3 (211.3–577.0 mg L^−1^), B-7 (65.0 mg L^−1^), Z-1 (350.2–1022.0 mg L^−1^), Z-2 (323.3–574.0 mg L^−1^), Z-3 (683.9 mg L^−1^), Z-4 (108.3–260.3 mg L^−1^) cores as well as samples collected from the lower layers of B-4 (128.0 mg L^−1^), B-6 (181.6 mg L^−1^) and B-8 (265.7 mg L^−1^) cores. Moreover, Cl^−^ levels in pore water samples taken from the second and third layers of the B-1 (277.4–303.9 mg L^−1^) and B-3 (100.9–105.1 mg L^−1^) cores were found to be higher than HCO_3_^−^ contents (31.7–95.6 mg L^−1^). The rest of the samples had higher contents of HCO_3_^−^ than those of Cl^−^. It is worth noting the pore water samples from the upper sediment layer at the B-8 location, whose HCO_3_^−^ (59.4 mg L^−1^) and SO_4_^2−^ (58.7 mg L^−1^) concentrations were almost similar, but Cl^−^ level was relatively low (4.2 mg L^−1^). In terms of chloride ion concentration, pore water samples can be divided into two categories: the first group (B-4, B-5, B-6, B-7, B-8, Z-1, Z-4) included the samples whose Cl^−^ levels (from 2.9 to 9.8 mg L^−1^) were similar to chloride ion concentration in the overlying water, and the second group (B-1, B-2, B-3, Z-2, Z-3) comprised samples which had higher chloride ion concentrations (from 15.3 to 303.9 mg L^−1^).

In all pore water samples, Ca^2+^ levels were higher than Mg^2+^, Na^+^ and K^+^ concentrations. Furthermore, all pore water samples had lower contents of K^+^ in relation to those of Mg^2+^ and Na^+^. The Mg^2+^ and Na^+^ concentrations were found to be almost similar amongst pore water samples taken throughout B-4 (6.0–13.1 and 8.5–10.3 mg L^−1^, correspondingly), B-7 (5.0 and 8.7 mg L^−1^, correspondingly), Z-3 (68.0 and 71.1 mg L^−1^, correspondingly), the uppermost layers of B-2 (13.0 and 15.3 mg L^−1^, correspondingly), B-6 (10.8 and 7.7 mg L^−1^, correspondingly), B-8 (6.5 and 9.6 mg L^−1^, correspondingly), Z-1 (13.3 and 10.1 mg L^−1^, correspondingly), Z-4 (8.9 and 9.0 mg L^−1^, correspondingly) and bottom layer of B-2 (32.4 and 34.8 mg L^−1^, correspondingly) cores. In the pore water samples taken throughout B-1 (67.0–144.0 mg L^−1^), B-3 (52.3–101.2 mg L^−1^), Z-2 (45.2–183.0 mg L^−1^) cores as well as from the second and third layers of the B-2 core (25.5–30.0 mg L^−1^), the levels of Na^+^ were higher than those of Mg^2+^. However, the concentrations of Mg^2+^ were higher in pore water samples taken throughout B-5 (15.0 mg L^−1^) and from bottom layers of B-6 (24.0 mg L^−1^), B-8 (18.0 mg L^−1^), Z-1 (28.0 mg L^−1^) and Z-4 (18.4 mg L^−1^) cores as compared with Na^+^ levels.

In flooded soil pore water samples, there was either an increase (B-5, B-7, Z-1, Z-2, Z-3) or a decrease (B-8, Z-4) in the concentrations of major ions in relation to the sediment pore water. In the flooded soil pore water samples from the B-5, B-8, Z-1, and Z-3 sites, the concentrations of each anion decreased in the following order SO_4_^2−^ > HCO_3_^−^ > Cl^−^; in samples from the Z-2 site, the anion concentrations followed the sequence SO_4_^2−^ > Cl^−^ > HCO_3_^−^ and in those from the B-7 and Z-4 locations, the correlation between the anions was as follows HCO_3_^−^ > SO_4_^2−^ > Cl^−^. Similar to the sediment pore water, the flooded soil pore water was dominated by Ca^2+^. In the flooded soil pore water samples from the B-7, B-8, Z-2, and Z-3 sites, the concentrations of major ions followed the sequence Na^+^  > Mg^2+^  > K^+^, while in samples at the B-5, Z-1, and Z-4 sites, these concentrations were Mg^2+^ > Na^+^ > K^+^.

As shown by the Piper diagram (Fig. 2), SO_4_^2−^ was a major anion in 23 samples; HCO_3_^−^ was a dominant anion in six samples of sediment and flooded soil pore water, and pore water samples at six locations had a mixed composition. At 28 locations, the major cation was Ca^2+^, seven samples had a mixed composition. In the area under study, 12 locations represented SO_4_–Cl–Ca–Mg water type, six samples were of HCO_3_–Ca–Mg type and 17 locations represented a mixed water type.

## Discussion

The results reveal that only minimum Cl^−^ concentration in sediment pore water in the Bratsk reservoir was similar to its average content in the overlying water (Table 2). In pore water samples, minimum concentrations of HCO_3_^−^ were lower and those of SO_4_^2−^, Ca^2+^, Na^+^ and K^+^ were higher in relation to the overlying water. Maximum concentrations of major ions in the pore water were found to be higher than that of the overlying water by the following magnitude: HCO_3_^−^, 7 times; K^+^, Ca^2+^, and Mg^2+^, 11–26 times; SO_4_^2−^, Cl^−^, and Na^+^, 46–101 times. Therefore, the results indicate that variations in major ion concentrations between the pore and overlying water of the Bratsk reservoir are much greater than those obtained for the Ivan’kovskoe reservoir and Lake Baikal. Similar to the Bratsk reservoir, the overlying water in the Ivan’kovskoe reservoir is dominated by HCO_3_^−^ and Ca^2+^. In the Ivan’kovskoe reservoir, the increase in the concentrations of these ions in the pore water relative to their concentrations in the overlying water, occurs locally, in the area of the greatest anthropogenic impact^26^.

In contrast to overlying water, pore waters typically have low dynamic characteristics due to slow water exchange. It helped identifying the factors affecting the accumulation of chemical elements in the sediment pore water. Variations in ion concentrations between the overlying and pore water samples, combined with element concentrations in sediment pore water along the depth profiles, indicate that the chemical constituents of pore water in the Bratsk reservoir are affected by various factors as discussed below.

### Overlying water of reservoir

Earlier studies conducted on other water bodies have shown that the pore water of bottom sediments still retains some characteristics of the overlying water chemical composition. In the Caspian Sea, Cl^−^ concentrations determined in the pore and overlying waters were found to be similar and dependent on locations^32^. In the freshwater-saltwater transition zone, exemplified by the Yenisei River–Kara Sea profile, the pore water of the uppermost sediment layers exhibit a regular increase in the Cl^−^ concentration along the river mouth-open sea gradient^33^. Since Lake Baikal provides the major portion of the surface water to the Angara cascade reservoirs, the comparison of the Bratsk reservoir with the lake seems to be most appropriate. Earlier studies of bottom sediments from the pelagic zone of Lake Baikal, characterized by regular sedimentation rates^28^, demonstrated that similar to the lake waters, low-mineralized pore waters of Lake Baikal were dominated by calcium bicarbonate.

Initially, pore water is a reservoir bottom water trapped in particles during sedimentation. Therefore, at the initial stage of the sedimentation, the chemical compositions of overlying and pore water are similar. However, our results show significant variations in the chemical compositions of pore and overlying water with regard to major ions after 50 years of reservoir operation (Table 2). With regard to major ion composition, only the pore water taken from the upper part of the B-4 sediment core with the mineralization of 169 mg L^−1^ was similar to the overlying water.

The Bratsk reservoir is a reservoir with a high anthropogenic impact^34^. During the entire period of its operation, the hydrochemical composition was significantly influenced by the wastewater brought from the Usolie-Sibirskoe industrial zone. At present, the enterprises of chemical, pharmaceutical, food, and salt-mining industries, as well as machine-building plants and thermal electric power plants, are operating here. Significant amounts of Cl^−^, SO_4_^2−^, Na^+^, Ca^2+^, some lesser quantities of K^+^ and Mg^2+^ are brought into the Bratsk reservoir with the wastewater from the Usolie-Sibirskoe industrial zone^16^. Taken together, the results obtained from this study (Table S1, Fig. S1) confirm earlier conclusions^16^ stating that the technogenic migration of Cl^−^, SO_4_^2−^, Na^+^, Ca^2+^, and Mg^2+^ is mostly associated with their transport along the left bank of the reservoir. The levels of these ions decreased in the overlying water, reaching the mean values at a distance of 5 km downstream from the wastewater discharge system. The increase in the above ion concentrations in the overlying water close to Usolie-Sibirskoe town (Fig. S1) and their further decrease suggest that the anthropogenic factors have no profound effect on the accumulation of the major ions in the sediment pore water from the locations under study. At the same time, the Bratsk reservoir is characterized by a period with the maximum anthropogenic impact (1970–1998) and a period with a gradual decrease in the anthropogenic influence (from 1998 to present)^34,35^. These periods are related to the operation, gradual reduction and a subsequent deserting of the Usoliekhimprom enterprise, the largest enterprise of the Usolie-Sibirskoe industrial zone, which used to produce calcium carbide, caustic soda, calcium hypochlorite, sodium metal, etc. Therefore, we cannot completely exclude the impact of wastewater from the Usolie-Sibirskoe industrial zone on the concentration of major ions in the pore water.

### Sediments of reservoir

The terrigenous material, entering the reservoir due to the mechanical weathering of rocks and abrasions of shores, and transported with water flow, plays an important role in the genesis of pore water. The pore water chemistry is greatly influenced by the chemical composition of bottom sediments. It was earlier shown that pore water contained in sediments have higher CaCO_3_ (> 90%) contents, demonstrated pH values ranging between 7.7 and 8.2, relatively low TDS values and represented HCO_3_–Ca, HCO_3_–Ca–Mg or HCO_3_–SO_4_–Ca–Mg hydrochemical water types^36^.

In the Bratsk reservoir bottom sediments, the medium-grained fraction amounted from 0.1 and to 10.2%, fine-grained—from 1.3 and to 39.5%, the coarse-dust fraction—from 4.5 and to 79.4%, fine-dust fraction—from 1.5 and to 78.1%, and clay fraction—from 1.7 and to 46.2%. A total of 16 samples of bottom sediments were classified as silty-clayey, 8 samples as sandy loam, 3 samples as clays, 1 sample as sand (Figs. 3, 4).

The most common rocks in the catchment area are carbonates and sulfates, including dolomites, limestones (calcite), gypsum, and anhydrites (Fig. 1c). The composition of the Bratsk bottom sediments on the site from Svirsk to Unga Bay revealed that the total content of carbonates, with a predominance of CaCO_3_ and MgCO_3_, averaged to 24%^19^. With time, the sediment-forming terrigenous material underwent leaching that resulted in the increase of Ca^2+^, Mg^2+^, HCO_3_^−^ and SO_4_^2−^ concentrations in the Bratsk reservoir pore water.

Within the channel part, the highest Ca^2+^, Mg^2+^ and SO_4_^2−^ concentrations were recorded in the sediment pore water samples from the B-1 and B-3 locations, marked by high sedimentation rates. As follows from the regional geology (Fig. 1c), the main component of bottom sediments from these locations is terrigenous material coming from the abrasion of shores, composed of easily eroded gypsum-salt-bearing-carbonate rocks. The gypsum-bearing facies present amongst the sedimentary strata resulted in water with high concentrations of sulphate and calcium. At locations B-1, B-2, B-3, the sediment composition is greatly influenced by the Angara River and its tributaries—the Irkut, Kitoy, Belaia rivers, carrying the minerals formed from breaking apart of sedimentary rocks (calcite, halite, clay minerals) and igneous Archaean rocks (quartz, feldspars, mica)^13^. Although the pore water samples at sites B-1, B-2, and B-3 were produced in a single geologic zone, SO_4_^2−^, Ca^2+^ concentrations and the pore water mineralization at B-2 were lower, which might result from a higher proportion of quartz, feldspars, and clay minerals (alumosilicates and silicates) at B-2 in relation to B-1 and B-3 sites.

In the studied pore water samples, the major ions showed maxima in the entries of Osa, Unga and Tal'kino bays (Figs. 3, 4). At locations Z-1 and Z-2, the sediments have a greater share of the material transported from the catchment areas of the Reservoir’s tributaries—the Unga, Zalarinka and Osa rivers. At the confluence of the Bratsk Reservoir, gypsum-bearing rocks of the Upper Lena Formation occurring in the catchment area (Fig. 1) play a dominant role in SO_4_-Ca type water formation with mineralization up to 1112 mg L^−1^ in the Unga River, to 1127 mg L^−1^ in the Zalarinka River and up to 414 mg L^−1^ in the Osa River.

The mineralization of the pore water at locations B-5, B-6, B-7, B-8, and Z-4 was much lower in relation to B-1, B-2, B-3, Z-1, Z-2, and Z-3 sampling sites. First, this can be explained by lower sedimentation rates and, therefore, better conditions for circulation between low-mineralized overlying and pore water at B-4, B-5, B-6, B-7, B-8 and Z-4. At B-5, B-6, B-7, B-8, and Z-4 sampling sites, the sediments were produced with a greater share of the red-color terrigenous-carbonate formations. Earlier mineralogical studies show that the sediments include marls (pelite material with the carbonate cement), mudstones (clayey material composed of alumosilicates with higher contents of calcium and iron oxides), and siltstones (unrounded and angular grains; the spaces between grains are filled with clay-iron oxide cement, which contains calcite or dolomite^12^. At these locations, together with sampling sites with higher sedimentation rates, the increase in concentrations of major ions, primarily Ca^2+^, Mg^2+^ and HCO_3_^−^ in the pore water can be explained by the dissolution of the terrigenous material entering the sedimentation area.

In samples taken from both bottom sediments and flooded soils, a change in pore water mineralization is symbate. The pore water samples from soils and bottom sediments at Z-1, Z-2, and Z-3 locations had higher levels of major ions (Figs. 3, 4) in relation to Z-4, B-7, and B-8, indicating that bottom sediments generally inherited the composition of primary material of soil-forming rocks. In bays, this tendency was even more evident as the hydrodynamic conditions of a bay favored the deposition of a larger portion of weathering products within its water area. Accumulation of bottom sediments at Z-1 and Z-2 locations was greatly influenced by the sulfate karst formation, in which displacements occurred along the clay interlayer formed at the contact between gypsum-anhydrite rocks and layers of limestones and gypsum-bearing dolomites^37^. The karst processes are particularly intense in Osa Bay^38^. In the Bratsk reservoir, the intense karst-landslide deformations under water level changes^12^ are one of main factors triggering the accumulation of terrigenous sulfate formation in bottom sediments.

**Figure 5:**
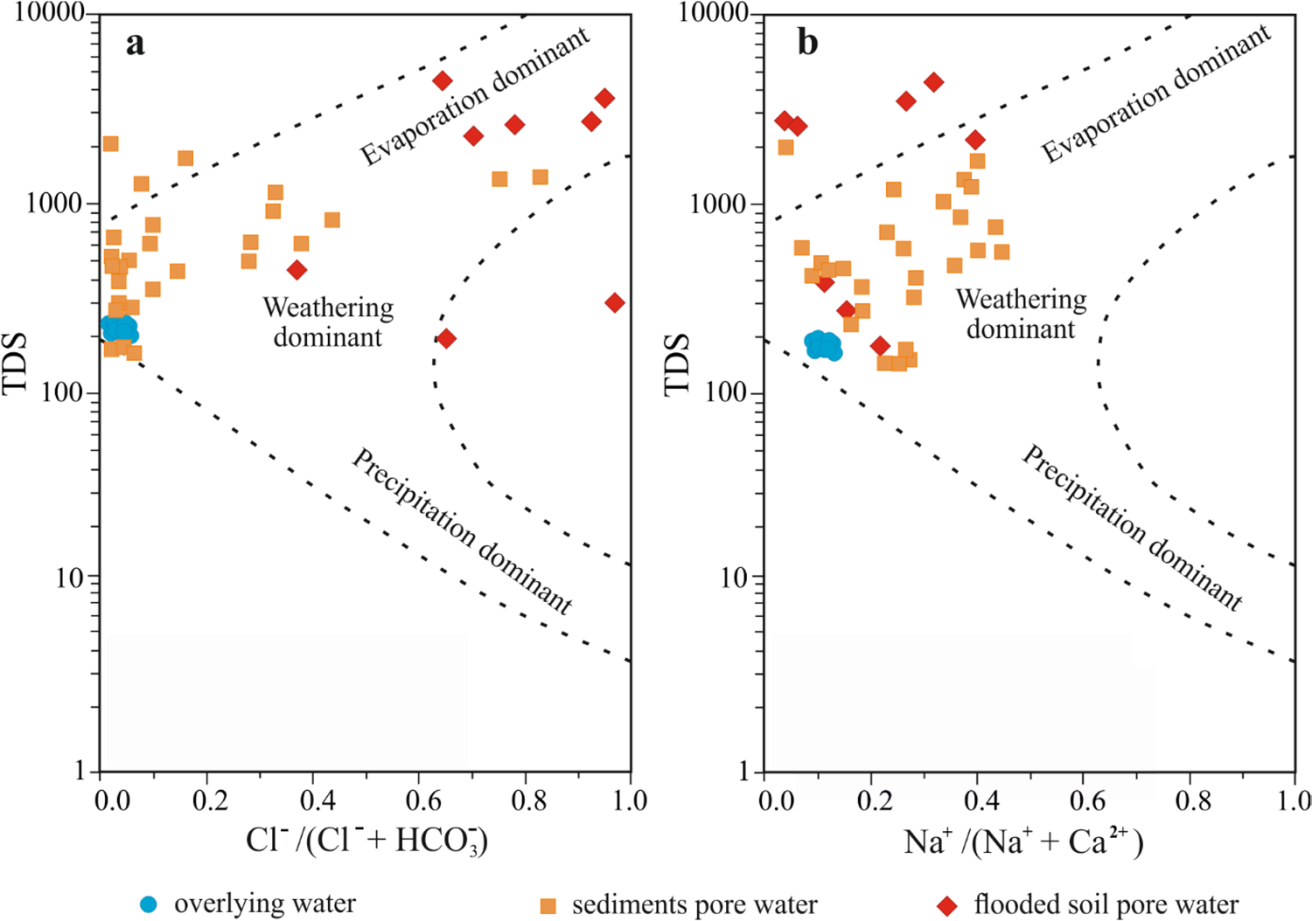
Gibbs diagram of overlying and pore water: (**a**) total dissolved solids (TDS) vs. equivalence ratio of Cl^−^/(Cl^−^ + HCO_3_^−^); (**b**) TDS vs. equivalence ratio of Na^+^/(Na^+^ + Ca^2+^). Figures were created using Grapher (17.2.435, www.goldensoftware.com) and CorelDRAW (21.3.0.755, www.corel.com).

Gibb’s diagram is applied to discriminate the leading factors that are responsible for the water chemical composition, such as precipitation, weathering processes, and evaporation^39^. On the Gibb’s diagram, all samples of surface and bottom water fall mainly in the water–rock interaction zone (Fig. 5). The majority of pore water samples fall in the rock dominant zone, suggesting that the concentrations of major ions in pore water were mainly controlled by the dissolution of minerals in the sediment matrix (i.e. dolomite, limestone (calcite), gypsum and anhydrite). Samples of flooded soil pore water at sites B-5, Z-1, Z-2, and Z-3 and samples of pore water from the second layer of bottom sediments at Z-1, Z-2 locations showed the inclination towards evaporation dominant zone. The same samples exhibited the highest concentrations of TDS values as well as elevated Cl^−^, Na^+^ and SO_4_^2−^. The source of Cl^−^ and Na^+^ in this pore water sample is saline soil, occurring around the Angara-Unga part of the Angara River Valley. Chloride ions, due to their high mobility, are easily introduced into pore waters and reach high concentrations. In the presence of salts, the CaSO_4_ solubility significantly increased.

**Figure 6:**
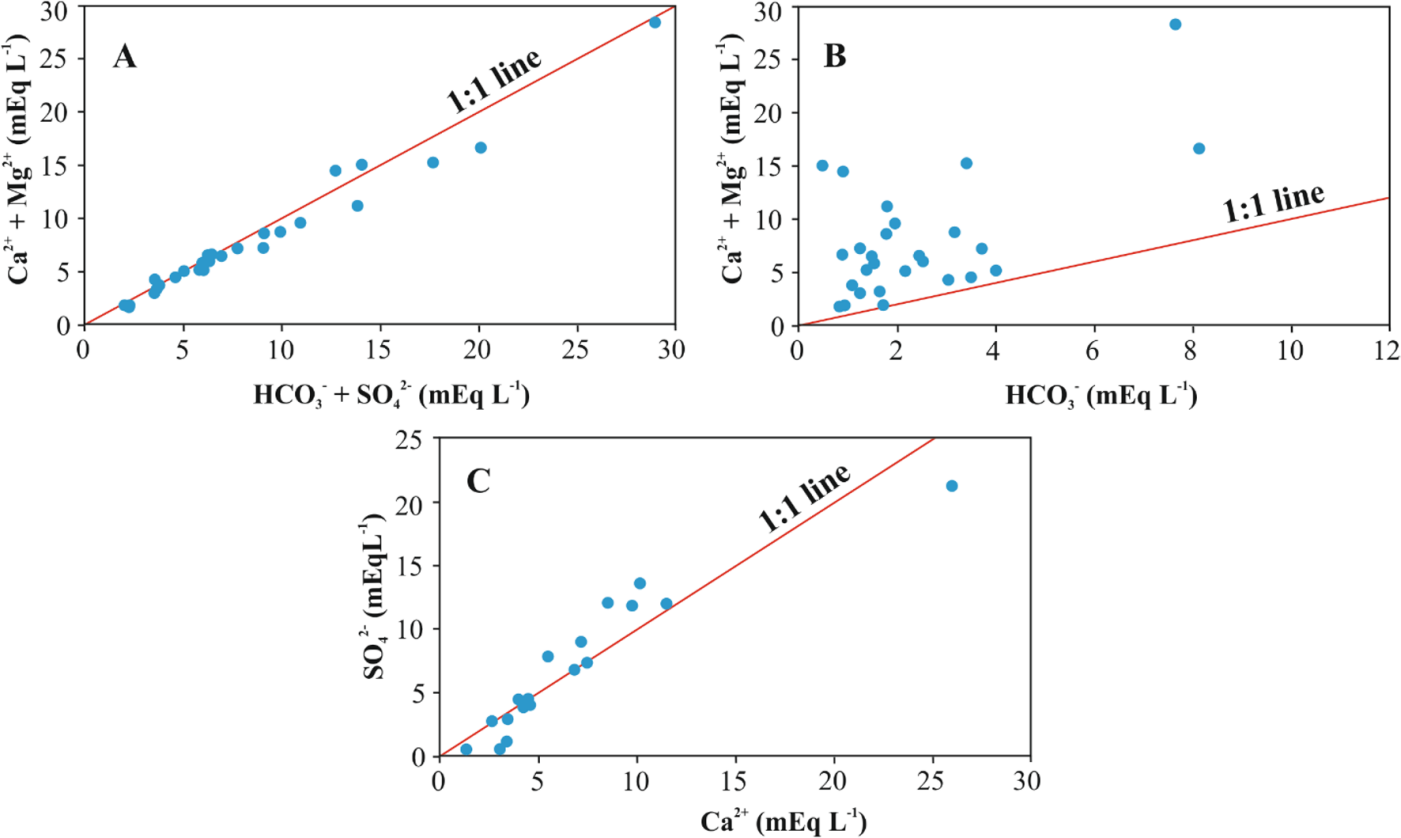
Distribution Ca^2+^ + Mg^2+^ vs HCO_3_^−^ + SO_4_^2−^ (**A**), Ca^2+^ + Mg^2+^ vs HCO_3_^−^ (**B**), SO_4_^2−^ vs Ca^2+^ (**C**). Figures were created using Grapher (17.2.435, www.goldensoftware.com).

The leaching processes were studied using scatter plots showing the enrichment of water with chemical elements due to the water–rock interaction^40^. The dominant reaction, occurring in the pore water-bottom sediment system, is demonstrated by a scatter plot between (Ca^2+^ + Mg^2+^) vs (SO_4_^2−^ + HCO_3_^−^) (Fig. 6A) showing the dissolution of calcite, dolomite, anhydrite and gypsum. Most pore water samples occurred along the 1:1 equiline (Fig. 6A). Moreover, if ion exchange is the dominant process, the data points of the plot tend to shift to the right due to the excess of HCO_3_^−^ + SO_4_^2−^ over Ca^2+^ + Mg^2+^. However, if the dominant process is reverse ion exchange, the points are shifted to the left^41^.

Figure 6B shows that the dissolution of carbonate rocks is not the major process influencing pore water chemistry within the study area. In groundwater samples, Ca^2+^ vs SO_4_^2−^ ratio which is close to the 1:1 equiline, implies that calcium and sulfate in water are products of gypsum and anhydrite weathering^42,43^. In the Ca^2+^ vs SO_4_^2−^ scatter plot (Fig. 6C), the majority of points of Bratsk reservoir pore water samples followed close to the 1:1 equiline, thus suggesting a significant input of sulfate rocks in the pore water chemistry. The points, corresponding to pore water samples, which occur above the 1:1 equiline, show that dissolutions of these minerals should occur, while excess calcium indicates an additional geochemical process^43^. In the Bratsk reservoir, this additional process included, importantly, dissolution of carbonates followed by cation exchange, during which the alkali and alkaline earth metals Na^+^, K^+^ and Mg^2+^ pass into the absorbing complex of the sediment and displace Ca^2+^ from this complex, leading to the increase of calcium levels in pore water.

In the Bratsk reservoir, the sedimentation rate is determined by the water level and velocity of the water flow. The most dynamic conditions are recorded in the variable backwater effect zone (flow velocity ranges from 0.57 to 0.24 m s^−1^) and the constant backwater effect zone (flow velocity from 0.24 to 0.09 m s^−1^)^13^. Downstream, the water flow velocity decreases, sometimes amounting to 0.01 m s^−1^ in the bottom layer. Changes in hydrodynamic parameters lead to erosion and the re-deposition of bottom sediments. The upper layer of bottom sediments is more dynamic, while the bottom sediments in the bottom part of the sediment core are inert and almost not affected by changes in hydrological conditions (especially in areas with higher sedimentation). Therefore, the concentrations of major ions in pore waters increase along the sediment depth profile (Figs. 3, 4). This is also facilitated by a longer period of interaction of pore water with the solid phase of the bottom sediment in the lower core layers at all sampling stations under consideration.

### Groundwater

Studies of hydrochemical characteristics in the Ust-Ilimsk and Boguchansk reservoirs, which are also parts of the Angara cascade, revealed that at several locations, the subaqueous groundwater discharge leads to increased concentrations of major ions in the bottom water relative to the surface one^44^. A lack of significant variations in the chemistry of surface and bottom waters within the Bratsk Reservoir (Fig. S1) shows that the groundwater chemical composition has a minor impact on the overlying water chemistry. As shown above, the increase in major ion concentrations in pore water relative to overlying water can be attributed to leaching processes. In addition, groundwater discharge, which is regarded as a synonym of pore water in water-saturated sediments^45^, could be regarded as an additional source of major ions in the pore water. In the view of Krivtsov and Sigee^46^, certain combinations of meteorological and hydrological parameters could cause groundwater percolation to the reservoir’s ecosystem, even through a low-permeable layer of clay deposits. Hence, percolation of more mineralization groundwater may be expected to affect the ionic composition of the sediment pore water. The impact of groundwater depends on the concentration gradient and groundwater volume.

As compared with the Angara River and lateral inflows, the groundwater discharge to the Bratsk reservoir is insignificant [not over 0.5 L (s·km^2^)^−1^]^47^. However, on the site from Usolie-Sibirskoe city to Unga and Osa bays, there is a network of discontinuous north-eastern and north-western faults^48^. The hydrogeodynamic and hydrogeochemical conditions suggest an upward movement of the artesian water flow with a head often exceeding the water levels in the Angara, Osa, and Unga rivers^12^. Before filling the Bratsk reservoir, fresh, hydrocarbonate alkaline earth groundwaters are widespread within sandy-clayey and carbonate sediments. At deeper aquifers, the hydrocarbonate groundwater composition changes into brackish sulfate and within gypsum sediments, they are changed into sulfate calcium^49^. After filling the reservoir, features of the tectonic structure of the coastal zone lead to a rise in the hypsometric levels of groundwater from deeper aquifers^12^.The greatest changes take place in the southern part of the reservoir (from Usolie-Sibirskoe town to Unga Bay): in the variable backwater effect zone, the pressure water is discharged as saline springs^47^, while on the Unga Bay coast, sodium chloride water with mineralization of 6.2 g L^−1^ has been found^49^. Here, a dome-like entry of mineralized groundwater from deeper aquifers (the so-called hydrogeochemical dome) into the upper mainly fresh-water section of the hydrogeochemical profile greatly influences the groundwater chemistry^49^. Sodium chloride water is the most common type in the centre of the hydrogeochemical dome, while brackish sulfate-type water occurs mainly in hydrogeochemical dome’s wings. As a result of annual and long-term water level fluctuations leading to changes in hydrogeodynamic conditions, there are continuous changes in groundwater heads^12^.

The results from hydrogeological studies suggest that at locations of intensive groundwater discharge into the reservoir’s bed, the concentrations of major ions in pore water can significantly increase. Such a trend was observed at B-1, B-3, Z-1, Z-2, and Z-3 sampling sites. Upon filling the reservoir, those sites demonstrated the extensive discharge of highly mineralized brackish sulfate and sodium chloride water. The hydraulic relations between ground and pore waters were found in the Selenga shallow water of Lake Baikal^50^. However, similar to the Bratsk reservoir, no influence of groundwater subaqueous discharge on bottom water mineralization has been found in Lake Baikal.

### Early diagenetic processes

Early diagenetic changes of a sediment begin after its deposition and include sediment compaction, mineral and chemical composition changes, and organic matter transformation that controls redox processes^51^. The early diagenetic transformations of Lake Baikal sediments were studied based on the distribution of major ions in sediment pore water^52^. It was found that, at sites with relatively calm sedimentation conditions (no faults, and groundwater infiltration), the pore water of the upper (oxidized) sediment layer contained much lower HCO_3_^−^ concentrations than the bottom water. In the reducing sediments of Lake Baikal, HCO_3_^−^ concentration increased due to the decay of the buried organic matter. On the contrary, the SO_4_^2−^conncentration showed the maximum in the oxidized layer, decreasing towards the bottom of the oxidized layer by an order of magnitude due to the bacterial sulfate reduction and remaining at this level to the bottom of the sediment core. In the Baikal region, a similar distribution of HCO_3_^−^ and SO_4_^2−^ was found in the organogenic sediments of small lakes^53^. As opposed to lakes with a longer existence period, early diagenetic transformations of sediments from the Angara cascade reservoirs, remains an insufficiently studied geochemical sector. However, the data obtained for the Bratsk reservoir show early diagenetic transformations of organic and mineral substances brought to the bottom of the Bratsk reservoir during sedimentation. In the Bratsk reservoir, one of the early diagenesis indicators is the change in the hydrochemical composition type of the sediment pore water in relation to the overlying water (Fig. 2, Table S2).

Negative Eh values in pore water samples of the Bratsk Reservoir (Figs. 3, 4) indicate that the bottom sediments undergo reduced diagenetic transformations whose intensity depends on the amount of organic matter^54^. In the Bratsk Reservoir bottom sediments, on the site from Svirsk to Unga Bay, the organic carbon content is on average, 5.60%, with its highest concentration in the bottom sediments of the bays (to 17.7%)^19^. It should be noted that large amounts of organic materials were brought during the reservoir filling when 166 thousand hectares of agricultural land and 135.2 thousand hectares of forest were flooded^22^. Under reducing conditions, the most mobile labile organic matter compounds are transferred to the solution due to microbiological transformations leading to an increase of HCO_3_^−^ contents in the pore water along the sediment depth profile.

The color of bottom sediments is thought to provide indirect information concerning the sediment composition, temporal dynamics of the sedimentation environment and the character post-sedimentation transformations^55,56^. In the Bratsk reservoir, the dynamics of diagenetic processes are shown by the visual color characteristics of sediments. Brown-orange color of the uppermost (0.5–1.0 cm) sediment layer, which interacts with oxygen-rich overlying water, implies the oxidative conditions. Gray and dark gray sediments, which are beneath brown-orange sediments, implies a change to reducing conditions.

In reducing sediments, the reduction of sulfate ions gives rise to sulfur compounds in a lower oxidation state. As follows from the Eh–pH sulfur diagram^57^, at Eh ranging between − 73 and − 346 mV and pH from 6.3 to 7.9, H_2_S and its dissociation product, hydrosulfide (HS^−^) ion should be present in Bratsk pore water samples (Fig. S2). At such pH values, sulfide ions in pore water are lacking. In sediments, free H_2_S is detected by smell^58^. In the bottom sediments of the Bratsk reservoir, the smell of hydrogen sulfide was recorded only in Tal’kino bay. It was found that the concentrations of SO_4_^2−^ in pore water samples increased as the sediment depth increased, thus suggesting that the sulfate reduction processes in Bratsk reservoir pore water were less intensive. Yudovich and Ketris^59^ showed that the poorly decomposed organic matter in anaerobic environments does not favor the processes of sulfate reduction and requires a longer presence of organic matter in anaerobic conditions. The major part of the organic carbon contained in the Bratsk sediments includes the humus substance^21^, which is highly resistant to the decomposition and to participation in biogeochemical transformations^60^.

## Conclusion

In the Bratsk reservoir, the chemical composition of pore water in the sediments showed significant variations across the water area and along the sediment depth profile. It was found that the initial pore water composition was primarily determined by the HCO_3_–Ca-type overlying water. During the sedimentation, the pore waters were characterized as HCO_3_–SO_4_–Ca, SO_4_–Cl–Ca–Mg and mixed water types. The pore water mineralization of the cored sediments displayed a clear increasing trend with depth. Negative Eh values of pore water samples and the color characteristics of the sediments showed that the bottom sediments of the Bratsk reservoir were subject to regenerative diagenesis. The concentrations of sulfates in the pore water samples revealed that the intensity of their reduction to sulfides was not high.

The present study revealed that, during a 50-year period of the reservoir’s operation, the concentrations of the major ions, mainly Ca^2+^, Mg^2+^, HCO_3_^−^ and SO_4_^2−^ in the pore water were primarily due to the dissolution of the sulfate-carbonate sedimentary material. However, some pore waters may have a complex genesis associated with subaqueous groundwater discharge, which is evidenced by higher Cl^−^, Na^+^ and SO_4_^2−^ concentrations in pore water from the south part of the reservoir.

The operation of the Bratsk reservoir, which results in interannual and seasonal fluctuations in the water level, leads to changes in the hydrological and hydrogeochemical conditions in its basin, the amounts of terrigenous material input, erosion, and re-deposition of bottom sediments, etc. Unstable sedimentation conditions inevitably affect the hydrochemical composition of pore waters. This is the first study to determine the factors affecting pore water chemistry in the Bratsk reservoir. In the future, studies aimed at microbial sulfate reduction processes, distribution patterns, and forms of trace elements in the solid phase and pore waters, their mineral composition will result in a more detailed approach to describing geochemical cycles of elements in a large reservoir.

## Supplementary Information

Supplementary Information 1.
