# Supplementary material for: Hydrochemistry of sediment pore water in the Bratsk reservoir (Baikal region, Russia)
Source: Sci Rep. 2021 May 27;11:11124. doi: 10.1038/s41598-021-90603-x (PMC8160327; doi:10.1038/s41598-021-90603-x)
Supplement: Supplementary file 1 — Supplementary Information 1. [file 41598_2021_90603_MOESM1_ESM.docx]

Supporting information for:

Hydrochemistry of sediment pore water in the Bratsk reservoir (Baikal region, Russia)

Vera I. Poletaeva*, Elvira N. Tirskikh, Mikhail V. Pastukhov

Vinogradov Institute of Geochemistry SB RAS, 1A Favorsky str., Irkutsk 664033, Russia

*Corresponding author

**
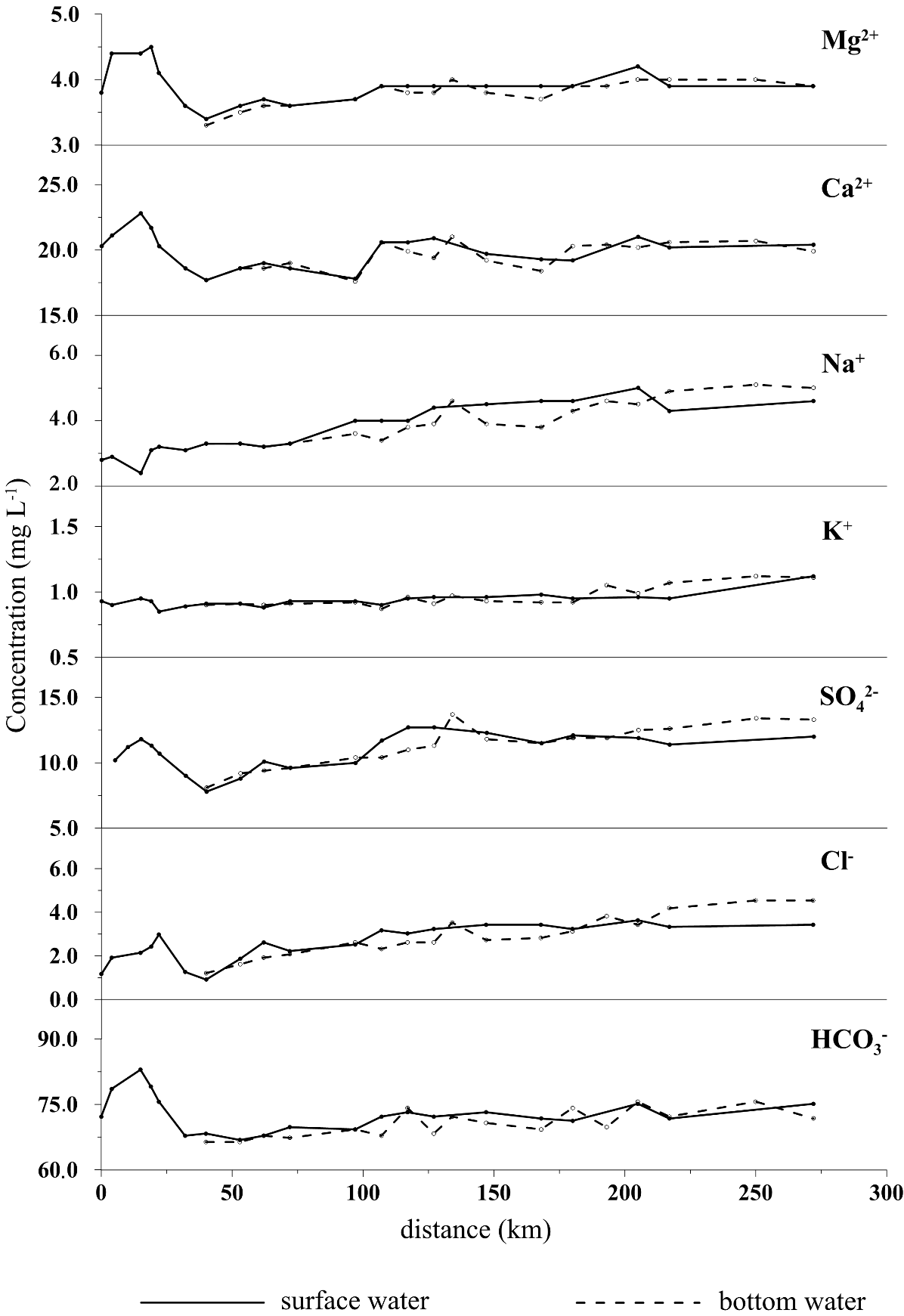
**

**Figure S1.** Distribution of major ions (mg L^-1^) in overlying water of the Bratsk reservoir. Main sampling sites: 0 km – 2 km above Usolie-Sibirskoe town, 7 km – entry of Usolie-Sibirskoe industrial zone wastewater, 16 and 20 km - 1.5 and 5 km downstream the entry of wastewater, 25 km – Belaya River mouth, B-1 – 53 km, B-2 – 62 km, B-3 – 72 km, B-4 – 107 km, Z-1 – 117 km, Z-2 – 134 km, B-5 – 147 km, B-6 – 168 km, Z-4 – 198 km, B-7 – 214 km, B-8 – 274 km. Figure was created using Grapher (17.2.435, www.[goldensoftware.com](file:///C:\Users\alieva\Documents\Статьи,%20тезисы\Статья_макро_поровые\Scientific%20Reports\После%20рецензии\Готовые\goldensoftware.com))

**
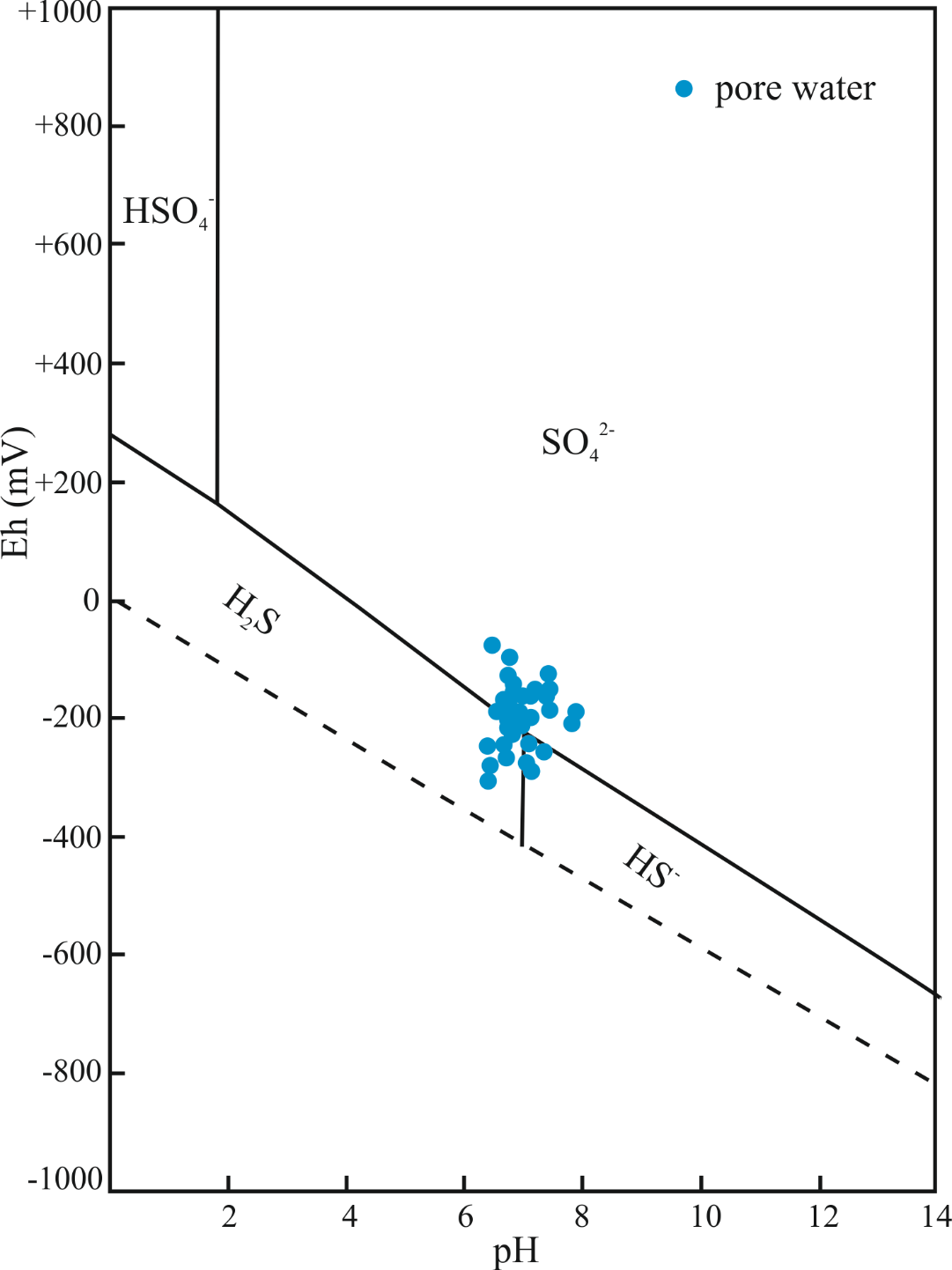
**

**Figure S2.** Eh-pH sulfur diagram of the Bratsk reservoir pore water. Figure was created using CorelDRAW (21.3.0.755, www.corel.com)

**Table S1**. Major ion concentrations (mg L^-1^) in overlying water, left bank of the Bratsk reservoir, Usolie-Sibirskoe industrial zone.

| Sampling site | HCO_3_^-^ | Cl^-^ | SO_4_^2-^ | Ca^2+^ | Mg^2+^ | Nа^+^ | K^+^ |
| --- | --- | --- | --- | --- | --- | --- | --- |
| Wastewater intake area | 87.4 | 18.0 | 15.4 | 25.3 | 5.3 | 11.6 | 0.93 |
| 1.5 km downstream of wastewater intake area | 84.4 | 8.8 | 13.5 | 24.3 | 5.0 | 6.9 | 1.05 |
| 5 km downstream of wastewater intake area | 96.1 | 4.9 | 10.1 | 25.9 | 5.0 | 4.3 | 0.96 |

**Table S2.** Pore water classification, Bratsk reservoir.

| Sampling site | | Type of water  by Kurlov ^a^ | Sampling site | | Type of water  by Kurlov | Sampling site | | Type of water  by Kurlov |
| --- | --- | --- | --- | --- | --- | --- | --- | --- |
| B-1 | 0-8  cm | $M_{621}\frac{\mathrm{HCO}_{3}14Cl16\mathrm{SO}_{4}20}{Na17Ca23}$ | B-4 | 0-10 cm | $M_{169}\frac{\mathrm{HCO}_{3}42}{Ca30}$ | Z-1 | 0-13  cm | $M_{643}\frac{\mathrm{SO}_{4}40}{Ca42}$ |
|  | 8-19  cm | $M_{1372}\frac{Cl18\mathrm{SO}_{4}31}{Na15Ca24}$ |  | 10-20 cm | $M_{282}\frac{\mathrm{HCO}_{3}14\mathrm{SO}_{4}34}{Mg13Ca31}$ |  | 13-26 cm | $M_{2073}\frac{\mathrm{HCO}_{3}13\mathrm{SO}_{4}37}{Ca45}$ |
|  | 19-30  cm | $M_{1331}\frac{Cl20\mathrm{SO}_{4}27}{Na15Ca24}$ | В-5 | 0-17 cm | $M_{462}\frac{\mathrm{SO}_{4}16\mathrm{HCO}_{3}33}{Ca34}$ |  | 26-46 cm | $M_{2753}\frac{\mathrm{SO}_{4}42}{Ca45}$ |
| B-2 | 0-8  cm | $M_{386}\frac{\mathrm{HCO}_{3}35}{Ca33}$ |  | 17-34 cm | $M_{2668}\frac{\mathrm{SO}_{4}46}{Mg11Ca37}$ | Z-2 | 0-16 cm | $M_{766}\frac{\mathrm{HCO}_{3}15\mathrm{SO}_{4}30}{Ca32}$ |
|  | 8-19 cm | $M_{359}\frac{\mathrm{HCO}_{3}34}{Ca28}$ | B-6 | 0-8  cm | $M_{283}\frac{\mathrm{HCO}_{3}22\mathrm{SO}_{4}25}{Mg12Ca31}$ |  | 16-32 cm | $M_{1730}\frac{\mathrm{HCO}_{3}17\mathrm{SO}_{4}24}{Na16Ca24}$ |
|  | 19-30 cm | $M_{433}\frac{\mathrm{HCO}_{3}18\mathrm{SO}_{4}23}{Mg13Ca27}$ |  | 8-17 cm | $M_{486}\frac{\mathrm{HCO}_{3}19\mathrm{SO}_{4}29}{Mg14Ca31}$ |  | 32-42 cm | $M_{3663}\frac{\mathrm{SO}_{4}40}{Na18Ca25}$ |
|  | 30-41 cm | $M_{631}\frac{\mathrm{HCO}_{3}21\mathrm{SO}_{4}23}{Mg15Ca26}$ | В-7 | 0-7  cm | $M_{166}\frac{\mathrm{HCO}_{3}18\mathrm{SO}_{4}29}{Ca30}$ | Z-3 | 0-20  cm | $M_{1262}\frac{\mathrm{SO}_{4}39}{Mg15Ca26}$ |
| B-3 | 0-8 cm | $M_{512}\frac{\mathrm{SO}_{4}30}{Na15Ca27}$ |  | 7-16 cm | $M_{304}\frac{\mathrm{SO}_{4}14\mathrm{HCO}_{3}34}{Ca36}$ |  | 20-45 cm | $M_{3611}\frac{\mathrm{SO}_{4}44}{Mg17Ca24}$ |
|  | 8-21 cm | $M_{607}\frac{Cl16\mathrm{SO}_{4}25}{Na20Ca23}$ | В-8 | 0-6  cm | $M_{169}\frac{\mathrm{HCO}_{3}21\mathrm{SO}_{4}27}{Ca28}$ |  | 45-70 cm | $M_{4608}\frac{\mathrm{SO}_{4}43}{Mg17Ca23}$ |
|  | 21-42 cm | $M_{799}\frac{Cl13\mathrm{SO}_{4}33}{Na19Ca24}$ |  | 6-14 cm | $M_{460}\frac{\mathrm{SO}_{4}43}{Ca36}$ | Z-4 | 0-8 cm | $M_{258}\frac{\mathrm{HCO}_{3}17\mathrm{SO}_{4}31}{Ca33}$ |
|  | 42-64 cm | $M_{932}\frac{\mathrm{SO}_{4}33}{Na16Ca26}$ |  | 14-32 cm | $M_{196}\frac{\mathrm{SO}_{4}37}{Ca30}$ |  | 8-16 cm | $M_{496}\frac{\mathrm{SO}_{4}39}{Ca35}$ |
|  | 64-86 cm | $M_{1096}\frac{\mathrm{SO}_{4}37}{Na14Ca27}$ |  |  |  |  | 16-24 cm | $M_{450}\frac{\mathrm{SO}_{4}23\mathrm{HCO}_{3}26}{Ca35}$ |

^a^  The Kurlov formula [1] is used to visualize the data on the chemical composition of pore waters. The Kurlov formula is a pseudo-fraction, the numerator of which represents the anions and the denominator designates cations given as a percentage of the amount of the equivalent substance.

[1] - Alekin, O.A. Principles of hydrochemistry [in Russian]. (Hydrometeorologicheskoe Izdat, Leningrad, 1970). – M – mineralization (mg L^-1^); the number next to the ion mean the ion content (% mEq L^-1^) of the total number of cations and anions corresponding to 100%. The type of water is determined by ions with the content more than 12%.
